# Biochemical Characterisation and *in vitro* Antitumour Effect of Parotoid Gland Secretions of the Egyptian Toad (*Bufo relgularis*)

**DOI:** 10.21315/tlsr2024.35.2.4

**Published:** 2024-07-31

**Authors:** Sabry Ali El-Naggar, Mohamed Aboulfotouh Basyony, Hany M. El-Wahsh, Seham Mohamed El-Feki, Ramadan Mahmoud Kandyel

**Affiliations:** 1 Zoology Department, Faculty of Science, Tanta University, Al-Geish St., Tanta, Gharbia 31527, Egypt; 2 Marine Biology Department, Faculty of Marine Sciences, King Abdulaziz University, Jeddah 21589, Saudi Arabia

**Keywords:** Parotoid Gland, Secretions, Antitumour, Apoptosis, Cell Cycle

## Abstract

This study aims to determine the biochemical compositions and the *in vitro* antitumour effect of the parotoid gland secretions (PGS) of the Egyptian toad (*Bufo regularis*). The total protein, lipid, carbohydrate contents, total antioxidant capacity (TAC), the median inhibitory concentration (IC_50_) of 2,2-diphenyl-1-picrylhydrazyl (DPPH), sodium dodecyl-sulfate polyacrylamide gel electrophoresis (SDS-PAGE) profile, amino acid analysis, gas chromatography–mass spectrometry (GC–MS) analysis and minerals were determined in PGS. The *in vitro* antitumour effect of PGS against human hepatocellular carcinoma (HepG-2), breast adenocarcinoma (MCF-7) and normal lung fibroblast (WI-38) cell lines were determined. The total protein, lipid and carbohydrate contents of PGS were 250 ± 15 mg/g D.W, 33 ± 3.2 mg/g D.W and 5 ± 0.65 mg/g D.W, respectively, while its TAC was 16.56 ± 0.12 mg/g D.W and the IC_50_ of DPPH was 51.95 ± 2.95 mg/mL. Six protein bands varied between 10 and 50 kDa were found in PGS. Among amino acid profile, arginine showed the highest content in PGS. GC-MS analysis showed that 11-octadecenoic acid methyl ester was the highest concentrations in PGS. The half-maximal inhibitory concentrations (IC_50_) of PGS against HepG-2, MCF-7 and WI-38 cells were 131.82 ± 6.14, 189.71 ± 8.95 and 685.65 ± 33.1 μg/mL, respectively. *In vitro* study showed that treatment of HepG-2 and MCF-7 cells with PGS increased the percentages of early and late apoptotic. While the percentages of early and late apoptotic WI-38 cells after treatment with PGS were 2.1% and 3.7%. Cell cycle analysis showed that PGS treatment arrested HepG-2 and WI-38 in S-phase, while arrested MCF-7 cells in G2/M phase. The present study concluded that PGS has a potent antioxidant activity with *in vitro* antitumour effect against HepG-2 and MCF-7 cells.

**Highlights**

- Biochemical analysis of parotid gland secretions (PGS) showed that it has a high protein content and possible antioxidant properties.
- The PGS revealed the presence of 17 amino acids, six protein bands by sodium dodecyl-sulfate polyacrylamide gel electrophoresis (SDS-PAGE)analysis and nine chemical compounds by gas chromatography–mass spectrometry (GC–MS) detection.
- The PGS showed strong anticancer efficacy against human hepatocellular carcinoma (HepG-2) and breast adenocarcinoma (MCF-7) cells *in vitro*.

## INTRODUCTION

Cancer is a complicated, multifaceted cellular disorder. Cancer is the second leading cause of mortality after cardiovascular disease in almost 130 countries worldwide (Bray *et al*. 2021). In 2020, the coronavirus disease (COVID-19) pandemic had a negative impact on cancer detection and therapy (Nnaji & Moodley 2021). By 2030, it is projected that there will be 17 million cancer deaths and 26 million new incidences of cancer per year (Allemani *et al*. 2018). Chemotherapy is currently one of the most vital and fundamental cancer treatments. However, it induces a variety of negative effects on vital organs in cancer patients (El-Naggar *et al*. 2011). It has been shown that natural products can be useful for creating novel medications (Atanasov *et al*. 2021). Typically, plants are the main source of these compounds, in addition, microorganisms, venomous and poisonous animals could be novel sources for finding antitumour agents (El-Naggar *et al*. 2015; Atanasov *et al*. 2021; Salama & El-Naggar 2021). Several studies have investigated new synthetic compounds or natural agents as pharmacological models for antitumour activity (El-Naggar *et al*. 2020; 2022).

Amphibian-derived compounds are among the most significant sources of molecules with bioactive properties from other species (Kowalskia *et al*. 2018). According to their new way of life and on the basis of adaptability, these animals’ skin secretions were altered (Ailin *et al*. 2019). The parotoid glands of the genus *Bufo* are situated on the sides of the head, as well as the neck or shoulder areas (Perry 2000). Proteins, peptides, poisonous substances, steroids, alkaloids and biogenic amines are among the substances released by these glands (Rodriguez *et al*. 2020). They have been widely utilised in America, Asia and Europe as antiviral, antibacterial, antidiabetic, anticancer, anti-inflammatory and analgesic properties (Neerati 2015; Da Mata *et al*. 2017).

Parotoid gland secretion (PGS) released from the skin involved in defense against predators and germs (Kowalskia *et al*. 2018). Currently, no available studies are reported on the chemical composition of and biological effects of PGS of the Egyptian toad *B. regularis*. However, the PGS compositions of South American toads were found to included alkaloids, argininyl diacids, bufadienolides and argininyl diacid derivatives from bufadienolides (Schmeda-Hirschmann *et al*. 2017). The composition of PGS obtained from *Rhinella horribilis* contains argininyl diacids, the alkaloid guanidinosuccinic acid, dehydrobufotenine, bufadienolides such as bufalin, gamabufotalin and argininyl diacids of bufadienolides (Schmeda-Hirschmann *et al*. 2020). Furthermore, the chemical composition of PGS of the Amazonian toad *Rhinella margaritifera* showed presence of arginine diacids, six bufagenins (telocinobufagin, marinobufagin, bufotalin, cinobufotalin, bufalin and cinobufagin), six bufotoxins and an alkaloid (dehydrobufotenin) (Sinhorin *et al*. 2020).

PGS of bufonid toads are a plentiful source of bioactive substances having cytotoxic and hemolytic effects (Kowalskia *et al*. 2018). The crude extracts of PGS of *Rhaebo guttatus* and *R. marina* showed strong toxic effects on rat breast carcinoma (Oliveira *et al*. 2019). PGS from Peruvian toad (*R. horribilis*) has anti-proliferative effect on lung cancer cell line (A549) (Schmeda-Hirschmann *et al*. 2020). In addition, peptides that isolated from PGS showed anticancer activity (Oelkrug *et al*. 2015). PGS could be unique resources for novel drug development. Therefore, this study was conducted to evaluate its biochemical compositions and its *in vitro* antitumour effect against two human cancer cell lines.

## MATERIALS AND METHODS

### Chemicals

2, 2 diphenyl-1-picrylhydrazyl (DPPH), methanol, sodium phosphate, ammonium molybdate, 3-(4, 5-dimethylthiazol-2-yl)-2, 5-diphenyl tetrazolium bromide (MTT), dimethyl sulfoxide (DMSO), cisplatin (Cis) and ascorbic acid were purchased from Sigma company (USA). Fetal bovine serum (FBS), Coomassie Blue Silver, dulbecco’s modified L-glutamine, gentamycin, HEPES buffer solution, DMEM (Eagle’s media) and 0.25% trypsin-EDTA were purchased from Lonza (Belgium).

### Collection of Toads and Preparation of PGS

Between November 2020 and January 2021, 100 toads (*B. regularis*) were collected from farms in Egypt’s Monufia Governorate’s Sadat City. The toads were carefully placed into wooden cages before being transported to the Tanta University Faculty of Science’s Zoology lab. The experiments were done in compliance with the guiding principles for the care and use of the laboratory animals at the Faculty of Science, Tanta University, under the ethical number (IACUC-SCI-TU-0244). Toads washed with saline, the latero-dorsal region of the parotoid macro-glands was gently squeezed by hand, and PGS were collected and put in a beaker. The toads were released to nature after sample collection. The collected secretions were lyophilised after being dissolved in distilled water, sonicated, stored at −20°C and dried under decreased pressure. The PGS were weighed and kept for further processing (Zoggel *et al*. 2012).

### Determination of The Total Protein, Lipid and Carbohydrate Contents

Total protein content was assessed using the technique of Waterborg and Matthews (1984). The total lipid content was determined according to Minotti and Aust (1987). Total carbohydrates were estimated according to the method of Cready *et al*. (1950).

### Determination of Total Antioxidant Capacity and DPPH Scavenging Activity

Total antioxidant capacity (TAC) was assessed using the phospho-molybednum technique according to Prieto *et al*. (1999). Spectrophotometric analysis was used to measure the PGS capacity to scavenge free radicals using the method of Blois (1958).

### GC-MS Analysis

PGS was prepared to determine different chemicals. Using a direct capillary column TG-5MS (30 m × 0.25 mm × 0.25 μm film thickness) and a Trace GC 1310-ISQ mass spectrometer (Thermo Scientific, Austin, TX, USA) according to El-Naggar *et al*. (2020).

### SDS-PAGE Analysis

SDS-PAGE (12% gel) examination of PGS was performed. Proteins bands were stained with Coomassie Blue Silver at 0.1%. In order to determine the molecular weights of proteins, a broad range molecular weights marker was run in parallel. The gel was then imaged, and the molecular weights were determined according to Laemmli (1970).

### Determination of Amino Acids Profile

To determine amino acids contents in PGS, briefly, 1 g of PGS was de-fated by diethyl ether and dried it in open air. Then 5 mL of 6 N HCl was added to sample and placed in oven at 110°C for 24 h. Take the tube out of the oven and let it 10 min. placed on filter paper and washed by adding 5 mL distilled H_2_O then incubated at water bath for 5 h for complete digestion. Excess H_2_O and HCl were then eliminated. PGS were dissolved in 2 mL buffer, filtered by 0.22 mm syringes. PGS diluted by dilution solution to 10% and then injected into amino acid analyser system (Wiedemair *et al*. 2020).

### Determination of Minerals Contents

The total of 8 mL of concentration HNO_3_ and 2 mL H_2_O_2_ were added to 500 mg PGS powder, then was digested by using microwave digestion system (Milestone, Ethos Easy model: ACT36). The digested sample then was diluted till 25 mL using deionised water. Serial dilutions were then prepared from a stock solution contains (1 g/L) of K, Mn, Fe, Mg, Zn, Na and Ca using deionised water. Different electrolytes standards were measured at ICP to get the standard curve (Perkin Elmer Model: Optima 700 DV). Using the WINLAB 32 software, samples concentrations were assessed as mg/dL (Dolan & Capar 2002).

### Cancer Cell Lines

Hepatocellular carcinoma (HepG-2), human breast cancer (MCF-7) and normal lung fibroblast (WI-38) cell lines were purchased from the American Type Culture Collection (ATCC) (Manassas, VA, USA). The cells were propagated in Dulbecco’s modified Eagle’s medium (DMEM) supplemented with 10% heat-inactivated fetal bovine serum (FBS), 100 U/mL penicillin, 100 mg/mL streptomycin and 100 mg/mL glutamine at 37°C in a humidified atmosphere containing 5% CO_2_. Cells were sub-cultured two to three times a week.

### *In vitro* Cytotoxic Effect by MTT Assay

The inhibitory concentration that kills 50% of cells (IC_50_) was determined by using MTT assay. Briefly, different concentrations of PGS were applied in triplicate to the MCF-7, HepG-2 and WI-38 cells at 70%–80% confluent, and the wells were incubated, then, each well received 10 mL of a stock solution of 12 mM MTT (5 mg/mL MTT in sterile PBS saline). After that, the sample was incubated at 37°C for 4 h. The purple formazan crystal that had formed at the bottom of the wells was then dissolved in 100 L of DMSO for 20 min after the MTT solution had been removed. Cis served as a positive standard. On an ELISA reader, at 570 nm, the absorbance was measured (StatFax-2100, Awareness Technology, Inc.). The sigmoidal curve was used to calculate the half-maximal inhibitory concentrations (IC_50_) that inhibit 50% of cells (Gomha *et al*. 2015).

### Determination of Apoptotic and Necrotic Percentages

Briefly, to determine the apoptotic percentage of PGS after 24 h exposure, cells were re-suspended at a concentration of 3 × 10^6^ cells/mL in 1X binding buffer. To 100 μL of cell suspension, 5 μL of Annexin-V and 5 μL of propidium iodide (PI) were added. At that time, the cells were gently shacked, then incubated for 15 min at room temperature (25°C) in the dark. 400 μL of 1X binding buffer were added, and a BD FACSC anto^TM^ II flow cytometer was used to examine the results (Eldehna *et al*. 2017).

### Cell Cycle Analysis

Cells from HepG-2, MCF-7 or WI-38 (2 × 10^4^/mL) were seeded and treated with IC_50_ of PGS for 24 h. The cells were then harvested and preserved in 70% cold ethanol overnight at 4°C. The fixed-cell pellets were obtained by centrifugation and re-suspended in PI/RNase staining Buffer after being washed with ice-cold PBS, and then analysed on a flow cytometer. Cell-cycle was calculated using CELLQUEST software (Becton Dickinson Immuno-cytometry Systems, San Jose, CA) (Eldehna *et al*. 2017).

### Statistical Analysis

Group’s data expressed as means ± S.D. were analysed by *t*-test while percentage data were analysed by SPSS software. *p* < 0.05 was considered as significance value for all statistical analyses in this study.

## RESULTS

### Macromolecule Contents, IC_50_ of DPPH Scavenging Activity and Total Antioxidant Capacity of PGS

**Figure 1:**
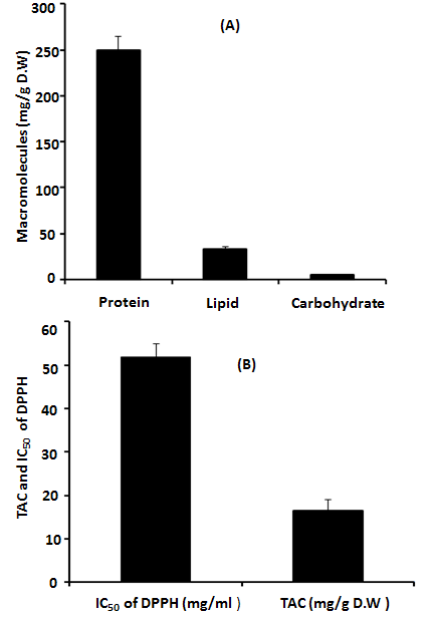
(A) The macromolecules content (total protein, lipid and carbohydrate) and (B)TAC and IC50 of DPPH in parotoid gland secretions.

The total of 100 Egyptian toads’ *B. regularis* were collected from Sadat City farms located in Monufia Governorate, Egypt. PGS were extracted and lyophilised to determine its macromolecules (total protein, lipid and carbohydrate) content. The total protein, lipid and carbohydrate contents were 250 ± 15 mg/g D.W, 33 ± 3.2 mg/g D.W and 5 ± 0.65 mg/g D.W, respectively (Fig. 1A). The total antioxidant capacity of PGS was 16.56 ± 0.12 mg/g D.W. The median inhibitory concentration (IC_50_) of DPPH was 51.95 ± 2.95 mg/mL (Fig. 1B).

### GC-MS Analysis of PGS

**Figure 2:**
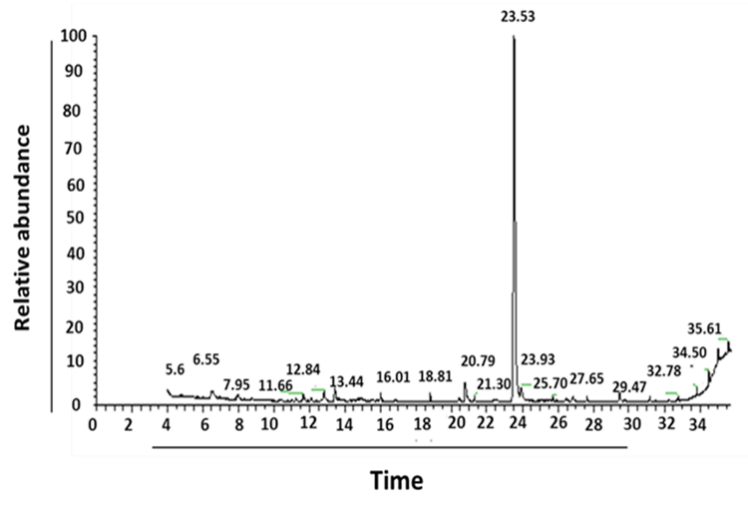
GC–MS chromatogram of parotoid gland secretions.

By GC-MS analysis of PGS, the chemical compounds were identified between retention time 6.51 min and 26.83 min. The results showed that there were nine chemical compounds with peak areas (PAs) varied from 0.89% to 70.12%. 11-octadecenoic acid, methyl ester represented the highest PA (70.12%), followed by hexadecanoic acid, methyl ester, 2-decyn-1-ol, cadinene, methyl stearate, β-ylangene, ss-caryophyllene and Z,Z,Z-4,6,9-nonadecatriene with PAs 3.92%, 2.43%, 2.34%, 1.90%, 1.77%, 1.63% and 1.22%, respectively. Cyclopentanetridecanoic acid, methyl ester represented the lowest PA (0.89%) (Fig. 2 and Table 1).

### SDS-PAGE, Amino Acids Profile and Minerals Content of Parotoid Gland Secretions

**Figure 3:**
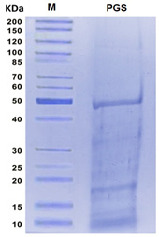
SDS-PAGE profile of parotoid gland secretions. Lane M = standard marker; Lane PGS = Parotoid gland secretions proteins; KDa = Kilodaltons.

**Figure 4:**
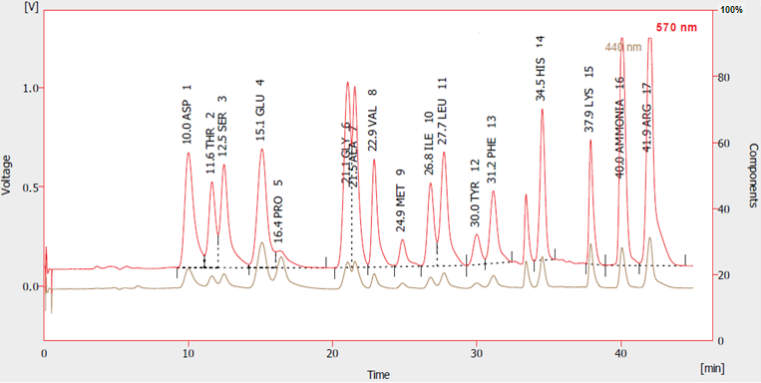
Amino acids chromatogram of PGS.

**Figure 5:**
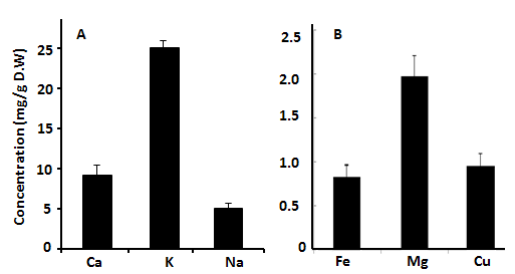
(A)The levels of Ca, K and Na; (B) Levels of Fe, Mg and Cu of PGS.

Quantitative analysis of proteins in PGS was performed using the SDS-PAGE gel. The results showed that presence of six protein bands ranging from 10 to ~50 kDa. The molecular masses of the protein bands were ~10, ~12.5, ~16, ~17.5, ~32.5 and ~50 kDa (Fig. 3). Between the retention times of 10.01 and 41.92, the amino acids were identified using an amino acid analyser. The findings revealed that arginine represented the highest concentration (23.2 mg/g D.W) followed by glutamate and aspartate, which represented 12.3 mg/g and 10.15 mg/g D.W, respectively, methionine represented the lowest concentration (1.86 mg/g D.W) (Fig. 4; Table 2). The results showed that potassium (K) represented the highest content, followed by calcium (Ca) and sodium (Na) levels in PGS (Fig. 5A). Iron (Fe) content represented the lowest concentration in PGS represented 0.85 ± 0.13. The content of Na, K, Ca, magnesium (Mg) and copper (Cu) were 5.05 ± 0.57, 25 ± 1.95, 9.2 ± 0.84, 1.98 ± 0.95, 0.23 ± 0.15 mg/g D.W, respectively (Figs. 5A and B).

### *In vitro* Cytotoxic Effect of Parotoid Gland Secretions Against HepG-2, MCF-7 and WI-38 Cell Lines

**Figure 6:**
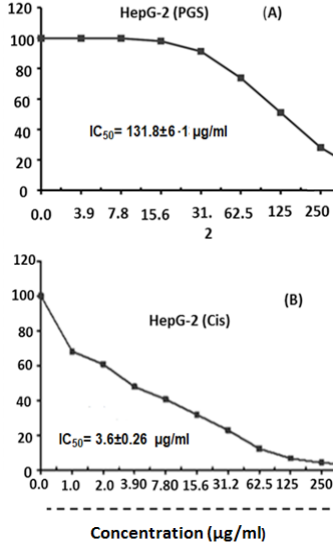
The half-maximal inhibitory concentration (IC_50_) of (A) parotoid gland secretions; and (B) Cis against HepG-2 cells.

**Figure 7:**
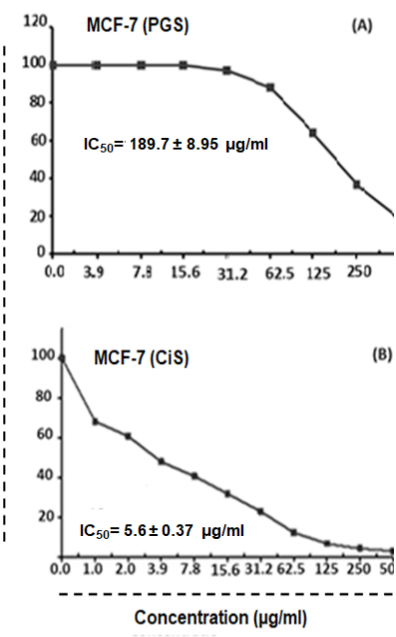
The half-maximal inhibitory concentration (IC_50_) of (A) parotoid gland secretions and (B) Cis against MCF-7 cells.

**Figure 8:**
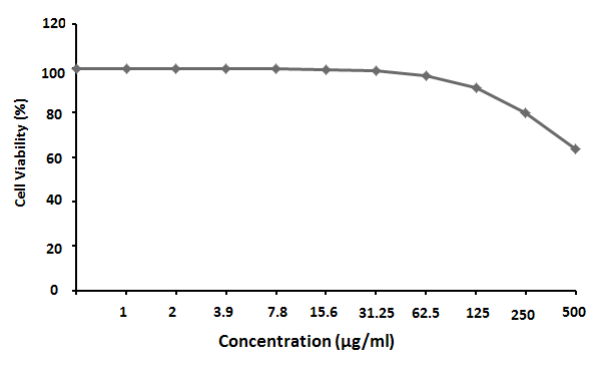
The half-maximal inhibitory concentration (IC_50_) of parotoid gland secretions against WI-38 cells.

The findings revealed that the IC_50_ of PGS against HepG-2 cells after 24 h was 131.82 ± 6.14 μg/mL. However, the IC_50_ of Cis against HepG-2 was 3.68 ± 0.26 μg/mL (Figs. 6A and B). The IC_50_ of PGS against MCF-7 cells after 24 h exposure was 189.71 ± 8.95 μg/mL and the IC_50_ of Cis against MCF-7 was 5.69 ± 0.37 μg/mL (Figs. 7A and B). While the IC_50_ of PGS against WI-38 after 24 h was 685.65 ± 33.1 μg/mL (Fig. 8).

### Effect of Parotoid Gland Secretions on The Percentage (%) of Apoptotic HepG-2, MCF-7 and WI-38 Cell Lines

**Figure 9:**
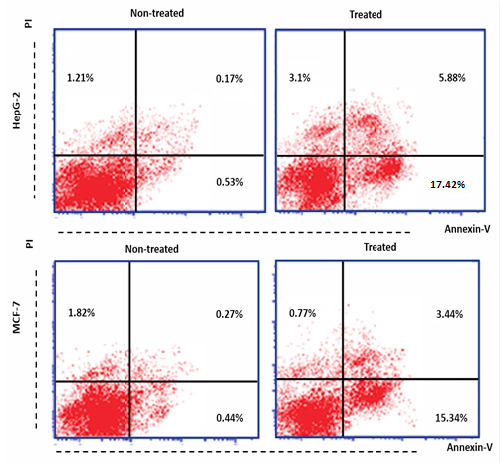
Apoptotic and necrotic percentages of the non-treated, treated HepG-2 and MCF-7 cells with PGS.

**Figure 10:**
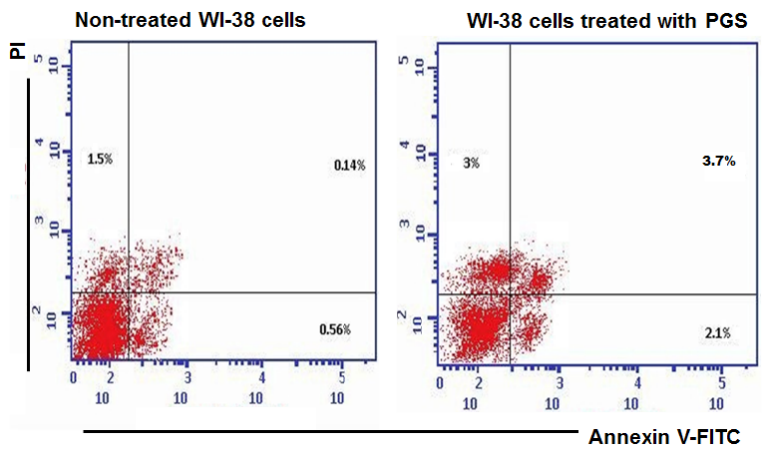
Apoptotic and necrotic percentages of the non-treated and treated WI-38 cells with PGS.

The findings indicated that the percentage of necrotic, early and late apoptotic of untreated HepG-2 cells were 1.21%, 0.53% and 0.17%, respectively. However, these percentages in the treated cells with PGS (1/10 of IC_50_) were 2.64%, 17.42% and 5.88%, respectively (Fig. 9). The percentage of necrotic, early and late apoptotic of untreated MCF-7 cells were 1.82%, 0.44% and 27%, respectively, however, after the treatment with PGS, these percentages were 0.77%, 15.34% and 3.44%, respectively (Fig. 9). While the percentage of necrotic, early and late apoptotic of non-treated WI-38 cell were 1.5%, 0.56% and 0.14%, respectively. However, these percentages in treated WI-38 cell with PGS were 3%, 2.1% and 3.7%, respectively (Fig. 10).

### Effect of Parotoid Gland Secretions on The Cell Cycle Phases of HepG-2, MCF-7 and WI-38 Cell Lines

**Figure 11:**
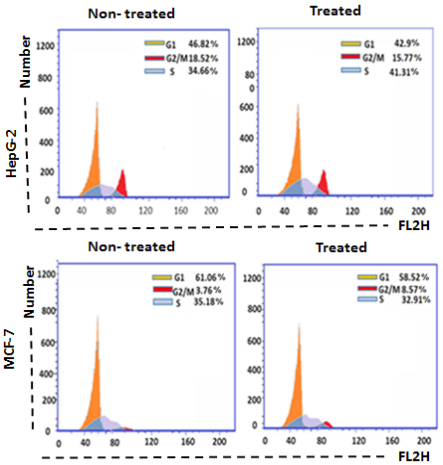
Cell cycle analysis of the non-treated, treated HepG-2 and MCF-7 cells with PGS.

**Figure 12:**
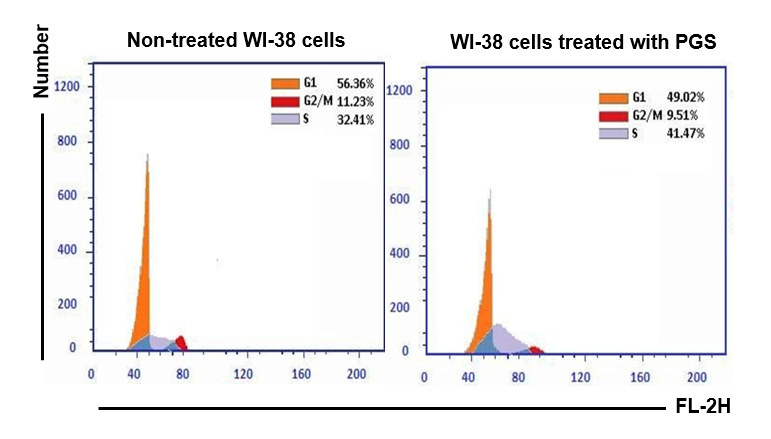
Cell cycle analysis of the non-treated and treated WI-38 cells with PGS.

The percentages of G1, S and G2/M in the non-treated HepG-2 cells were 46.82%, 34.66% and 18.52%, respectively. However, the treatment of HepG-2 cells with PGS for 24 h arrested the cell cycle in S phase (Fig. 11). The percentages of G1, G2/M and S phases in the non-treated MCF-7 cells were 61.06%, 3.76% and 35.18%, respectively. However, the treatment of MCF-7 cells with PGS for 24 h arrested the cell cycle in G2/M phase (Fig. 11). The percentages of G1, S and G2/M in the non-treated WI-38 cell were 56.36%, 32.41% and 11.23%, respectively. However, the treatment of WI-38 cells with PGS for 24 h arrested the cell cycle in S phase (Fig. 12).

## DISCUSSION

Amphibians are distributed worldwide, mainly in arid regions. Toads possess specialised macro-glands, identified as parotoids, behind the eyes and on their limbs (Ailin *et al*. 2019). In amphibians, the specialised skin macro-glands can secrete bioactive compounds (Rodriguez *et al*. 2020). Numerous compounds have been identified in PGS including peptides, steroids, indole alkaloids, bufogargarizanines and biogenic amines (Kowalskia *et al*. 2018). Several investigations purified and described the compounds on bufonid species physiologically and/or chemically (Schmeda-Hirschmann *et al*. 2016; Rodríguez *et al*. 2017). Therefore, these secretions may have anticancer properties (Lu *et al*. 2008).

Chemotherapy is used to treat cancer; however, complications can arise from its toxic nature (Nurgali *et al*. 2018). Alternative treatments that utilise molecules from amphibian skin secretions may be effective for cancer (Lu *et al*. 2008). PGS were extracted and lyophilised to determine their macromolecules content. The findings revealed that the total protein, lipid and carbohydrate contents of PGS were 250 ± 15 mg/g D.W, 33 ± 3.2 mg/g D.W and 5 ± 0.64 mg/g D.W, respectively. Previous study reported that the total protein concentration of toad poisons extracted from *Teresina* and *Picos* was 102.4 mg/g and 66.5 mg/g, respectively. Perry (2000) reported that PGS of *Amietophrynus* spp. contained a significant amount of protein (25%–35% by weight). Moreover, a previous report showed the presence of different types of lipids such as glycolipids, phospholipids and sterols in the PGS of *Bufo melanostictus* (Neerati 2015). The total antioxidant capacity of PGS was 16.56 ± 0.12 mg/g D.W and the DPPH scavenging activity was 96.25% with IC_50_ (51.95 ± 2.95 mg/mL). Previous study reported that skins from amphibians have potent antioxidant and free radical scavenging activities for their survival (Yang *et al*. 2009). By GC-MS analysis, several compounds such as 11-octadecenoic acid, methyl ester, hexadecanoic acid, methyl ester, 2-decyn-1-ol, cadinene, methyl stearate, β-ylangene, ss-caryophyllene and z,z,z-4,6,9-nonadecatriene were identified. Previous study by Schmeda-Hirschmann*et al*. (2016) reported that there were 29 compounds isolated and identified from the crude poison of *R*. *marina*. Major six protein bands with molecular masses of ~10, ~12.5, ~16, ~17.5, ~32.5 and ~50 kDa have been reported by SDS-PAGE profile. It has been reported that there was combination of proteins with relative molecular masses between around 12 to 200 kDa were present in the secretions of many species of *Bufo* by SDS-PAGE analysis (Perry 2000). deMedeiros *et al*.2019 reported that PGS of Amazonian *Rhinella marina* showed presence of protein bands in the apparent mass range of 6 to 195 kDa. A similar SDS-PAGE profile was found among the *Rhinella* species (Fontana *et al*. 2018). The amino acids profile of PGS showed that arginine represents the highest concentration followed by glutamate and aspartate; however, methionine represented the lowest concentration. These findings were in agreement with previous study who reported the detection of arginine amino acids at m/z 175 as the common amino acid in the PGS of Peruvian toad *Rhinella horribilis’* structural series (Schmeda-Hirschmann*et al*. 2020). Based on extensive search about the minerals content in PGS or similar skin secretions, so far, no available data about the levels of minerals have found in such these secretions. The K level represented the highest content of PGS, while the Fe represented the lowest concentration.

The IC_50_ of PGS against HepG-2, MCF-7 and WI-38 cells after 24 h were 131.82 ± 6.14 μg/mL, 189.71 ± 8.95 μg/mL and 685.65 ± 33.1 μg/mL, respectively. MTT assay showed that PGS have a cytotoxic effect against HepG-2 and MCF-7 cells. However, PGS has no toxic effect on WI-38 cells. These findings revealed that PGS has *in vitro* antitumour effect on HepG-2 and MCF-7 cells. These findings were in agreement with previous study which reported the *in vitro* antitumour effects of the PGS from the species of *Rhinella, Bufo* and *Rhaebo*, which have shown remarkable biological action on solid, sensitive and/or resistant human tumour cell lines (Sousa *et al*. 2017). This could be due to the presence of certain peptides in PGS that may have a cytotoxic effect on human cancer cells (Lu *et al*. 2008). Cinobufacin extracted from the dried skins of the toad *B. bufo gargarizans* displayed a dose-dependent inhibition on human hepatocellular carcinoma cell line (BEL-7402) and human gastric cancer cell lines (BGC-823) cells (Wei *et al*. 2017). Also, deMedeiros *et al*. (2019) showed the cytotoxic effects of crude venom of Amazonian *Rhinella marina* on human cells colorectal adenocarcinoma (HT29), colorectal carcinoma (HCT116), HepG-2, and lung normal fibroblast (MRC5).

The results showed that treatment of HepG-2 and MCF-7 cells with PGS increased the % of, early and late apoptotic. While no significant changes were found in these percentages in the treated WI-38 cells when compared to the non-treated cells. Previous study showed that toad skin extract of Chinese toad (*Bufo gargarizans*) induces apoptosis in HepG-2 cells (Lu *et al*. 2011). Another study showed that bufalin is an anticancer agent obtained from the skin and parotid glands of toads that could induce apoptosis in various lines of human tumour cells through its interaction with other genes and cellular elements (Takai *et al*. 2012). The protein BMP1 isolated from *B. melanostictus* inhibited cell proliferation and induced apoptosis on leukemic (U937, K562) and HepG-2 cells with minimum toxicity toward normal cell indicating its specificity (Gomes *et al*. 2011). The results showed that treatment of HepG-2 and WI-38 cells with PGS arrested cell cycle in S-phase. Previous study reported that Huachansu is a traditional Chinese medicine extracted from the skin of toads from the genus *Bufo* (*B. bufo gargarizans*) inhibited the growth of human gastric cancer (MGC-80-3) and hepatocellular carcinoma (SMMC-7721) cell lines through S-phase arrest (Meng *et al*. 2009). However, the treatment MCF-7 cells with PGS arrested cell cycle in G2/M phase. Furthermore, similar study reported that PGS of Peruvian toad *R. horribilis* induce apoptosis by halting the cell cycle before the G2/M-phase checkpoint (Schmeda-Hirschmann *et al*. 2020). Therefore, PGS has *in vitro* antitumor effect against HepG-2 and MCF-7 cells.

## CONCLUSION

This study reported the biochemical characterisation of the PGS that was obtained from the Egyptian toad (*B. regularis*) and showed its antitumour effect *in vitro* against two types of human cancer cell lines. We concluded that PGS has a potent antioxidant activity with *in vitro* antitumour effect.

## Figures and Tables

**Table 1:** Chemical compounds of parotoid gland secretions by GC/MS analysis.

No.	RT (min)	Compound name	MF	MW	PA (%)
1	6.51	2-decyn-1-ol	C_10_H_18_O	154	2.43
2	7.95	Z,Z,Z-4,6,9-nonadecatriene	C_19_H_34_	262	1.22
3	11.66	β-ylangene	C_15_H_24_	204	1.77
4	12.84	ss-caryophyllene	C_15_H_24_	204	1.63
5	13.43	Cadinene	C_15_H_24_	204	2.34
6	20.79	Hexadecanoic acid, methyl ester	C_17_H_34_O_2_	270	3.92
7	23.53	11-octadecenoic acid, methyl ester	C_19_H_36_O_2_	296	70.12
8	23.93	Methyl stearate	C_19_H_38_O_2_	298	1.90
9	26.83	Cyclopentanetridecanoic acid, methyl ester	C_19_H_36_O_2_	296	0.89

*Notes*: RT = retention time; MF = molecular formula; MW = molecular weight

**Table 2:** Amino acids analysis of parotoid gland secretions

No.	RT (min)	Name	Concentration (mg/g D.w)
1	10.01	Aspartate	10.15
2	11.64	Threonine	5.43
3	12.48	Serine	4.9
4	15.11	Glutamate	12.3
5	16.44	Proline	6.29
6	21.05	Glycine	8.2
7	21.54	Alanine	3.33
8	22.89	Valine	4.6
9	24.85	Methionine	1.86
10	26.80	Isoleucine	5.09
11	27.73	Leucin	6.43
12	30.00	Tyrosine	2.9
13	31.15	Phenyl alanine	5.36
14	34.54	Histamine	7.9
15	37.90	Lysine	4.8
16	40.03	Ammonia	5.49
17	41.92	Arginine	23.2

*Note*: RT = retention time
